# Experimental evaluation of the energy dissipation efficiency of the vortex flow section of drop shafts

**DOI:** 10.1038/s41598-023-28762-2

**Published:** 2023-01-30

**Authors:** Mohammad Mahmoudi-Rad, Mohammad Najafzadeh

**Affiliations:** 1 Department of Civil Engineering, Higher Education Complex of Bam, P.O. Box 76615314, Bam, Iran; 2 Department of Water Engineering, Faculty of Civil and Surveying Engineering, Graduate University of Advanced Technology, P.O. Box 76315116, Kerman, Iran

**Keywords:** Hydrology, Civil engineering

## Abstract

In urban wastewater collection and drainage networks, vortex structures are recruited to transfer fluid between two conduits with significant level differences. During the drop shaft, in addition to preventing the fluid from falling due to vortex flow formation, a significant amount of the fluid energy is dissipated due to wall friction of vertical shaft. In the present study, by constructing a physical model with a scale of 1:10 made of Plexiglas, the energy dissipation efficiency in the vertical shaft has been investigated. In this way, the performance of dimensional analysis indicates that the flow Froude number (*Fr*) and the ratio of drop total height to shaft diameter (*L⁄D*) are parameters affecting the efficiency of flow energy dissipation in the vertical shaft (*η*_s_). This research considers four levels of *Fr* factor (1.77, 2.01, 2.18, and 2.32) and three levels of *L⁄D* factor (10, 13, and 16). Additionally, four replications for 12 possible combinations allow us to carry out 48 experiments and the full factorial method. The results demonstrated that the energy dissipation efficiency in the vertical shaft changes varies from 10.80 to 62.29%. Moreover, *η*_s_ values decrease with an increase in *Fr* whereas the efficiency increases with increasing *L⁄D* ratio. Furthermore, the regression analysis gave a second-order polynomial equation which is a function of *Fr* and *L*⁄*D* to accurately estimate the flow energy dissipation efficiency in the vertical shaft.

## Introduction

In urban sewers and drainage systems, fluid transfer from the upper level to the lower level is occasionally performed through drop structures^1^. The two most common types of these structures are drop manholes and drop shafts^2,3^. Drop manholes are used to reach the goal of wasting energy and reducing the flow velocity at level differences of less than 7 m^4^ while drop shafts are used in level differences of more than 7–10 m^5^. Drop shafts are divided into two groups based on the type of flow formed: vortex drop shafts and plunging drop shafts^6,7^. Maintaining a steady flow pattern and wasting more energy for different discharges makes the vortex drop shafts superior to the drop shafts with plunging drop shafts^8,9^. The vortex structure consists of three main parts: the inlet structure, the vertical shaft, and the energy dissipater structure. The certain geometry of the inlet structure which is formed in various configurations such as spiral inlet^10,11^, tangential inlet ^8^, and scroll nlet^12^, is capable to convert the approach flow to vortex flow^13^. The vortex flow sticks to the vertical shaft wall to form an air core in column the center and moves downward annularly. Additionally, the friction of the wall is the most important factor in energy dissipation. This energy dissipation in the vertical shaft is mainly dependent on wall friction and turbulence of vortex flow. On the contrary, turbulence and air entrainment processes at the bottom of the drop shaft have no influence on the energy dissipation in the drop shaft^5^. In the outlet structure, in addition to the de-aeration, the remaining energy of the flow is highly dissipated^14^.

The vortex drop structures are basically employed in different fields. In this way, the most important practical examples are introduced as (*i*) municipal sewage of Milwaukee area with discharge of up to 90 m^3^/s over a drop height of 80 m^15^, (*ii*) the discharge of 140 m^3^/s and the high of 170 m, Curban, Italy^14^, (*iii*) power station of Shapai in China with a discharge of 200 m^3^/s and the drop height of about 100 m^16^, and (*iv*) discharge transmission of 1400 m^3^/s with the drop height of 190 m to the deviation tunnel in Xiaowan power stations in China^17^.

Generally, a considerable fraction of flow energy dissipation may be expected in the vortex drop shafts. Results of experimental research discovered that flow energy dissipation is likely due to the ratio of height to diameter (*L/D*) of the shaft. In the previous studies, the 85% and 90% fractions of flow energy dissipation have been estimated for the drop shaft with *L* = 50*D*^14^ and *L* = 100*D*^15^, respectively. These findings have been established based on the assumption that the drop shafts with a large height-to-diameter ratio may allow the flow to reach the terminal velocity. On the contrary, for drop shafts with a relatively small height-to-diameter ratio, 62% and 34% proportions of energy dissipation have been reported for *L* = 9*D*^18^ and for half of the length of a drop shaft with *L* = 14*D*^19^, respectively. Crispino et al.^20^ indicated that the energy loss efficiency was chiefly dependent on the flow effects and turbulence taking place in the chamber section. They proposed a relationship derived from a straightforward theoretical model in order to approximate the coefficient of energy loss. In case of dropshaft-tunnel systems, the experimental study performed by Chan and Chiu^21^ indicated that dropshaft Froude number and the ratio of dropshaft diameter to tunnel diameter had profound impacts on formation of various flow structures.

Design of experiments (DoE) involves methods that examine targeted effects on results (responses) by making targeted changes to one or more factors. In these methods, in order to achieve the response, the factors are tested simultaneously and the interactions among them are also considered. Referring to^22,23^, considering the design of experiments (i.e., full factorial), the purpose is to first show the possibility of investigating the main effective factors on the response of the physical model. For further explanation, it can be said that after selecting the various levels for each factor with consideration of the repetitions (at least two repetitions in order to calculate the error) for all possible combinations, the obtained results are analyzed using the ANOVA and the *p*-value statistical index. In recent years, formidable efforts have been made to investigate flow energy dissipation and mechanism of air entrainment in the vortex structures^22,23^. From these investigations, it can be proved that the successful usability of DOE in analysis of experimental research demonstrated optimum values of design dimensionless parameters when constructing vortex structures in the practical applications. In addition, the most recent investigation in which effects of dissipation chamber on the energy losses in the vortex energy was studied^24^. Mahmoudi-Rad and Najafzadeh^24^ found that the optimal values of effective parameters (i.e., *F*r and *L*/*D*) yielded 2.32 and 13.901, respectively; so as to stand the efficiency of flow energy loss at its highest level. Obviously, a great amount of flow energy in the vortex structures is dissipated in the vertical shaft section. Energy losses in the vertical shaft can be due to geometric properties of shafts and upstream approaching flow velocity to the inlet section of vortex structures. Under the aegis of the most relevant studies, only one effective factor (*i*.*e*., Froude number referring to hydraulic condition) has been investigated at several levels^14,15,18,19^ whereas *L*/*D* ratio was kept constant during investigations. The main differences between the present study and the other previous investigations on energy loss in the vertical shafts are that the simultaneous effect of two factors, introduced as hydraulic and geometric conditions, at a good many levels on the performance of the structure would be considered. In this way, the regression equation given to calculate energy loss of flow was dependent on the Froude number whereas geometric parameters of the structures have played a salient role in the estimation of losses. However, there is a fervid need for providing a mathematical model in order to predict the response of the shaft structure to the variations in hydraulic and geometric conditions. In the present study, the major drawback of the previous investigations has been resolved by considering the design of complete factorial tests for the vertical shaft.

The research organization of this study is presented as follows, (*i*) effective factors are introduced to estimate the flow energy dissipation efficiency in the vertical shaft of the vortex structure using dimensional analysis, (*ii*) the simultaneous effects of these factors on the *η*_s_ in the vertical shaft are investigated due to the limited use of experimental design in previous literature, (*iii*) experiments are designed and analyzed by full factorial method in order to evaluate the flow energy dissipation efficiency in the vertical shaft of vortex structure, (*iv*) a robust regression equation is given to describe how effective main factors are, and (*v*) the results of the present study are compared with relevant literature in terms of quantity and quality.

## Flow energy dissipation efficiency in the vertical shaft

**Figure 1:**
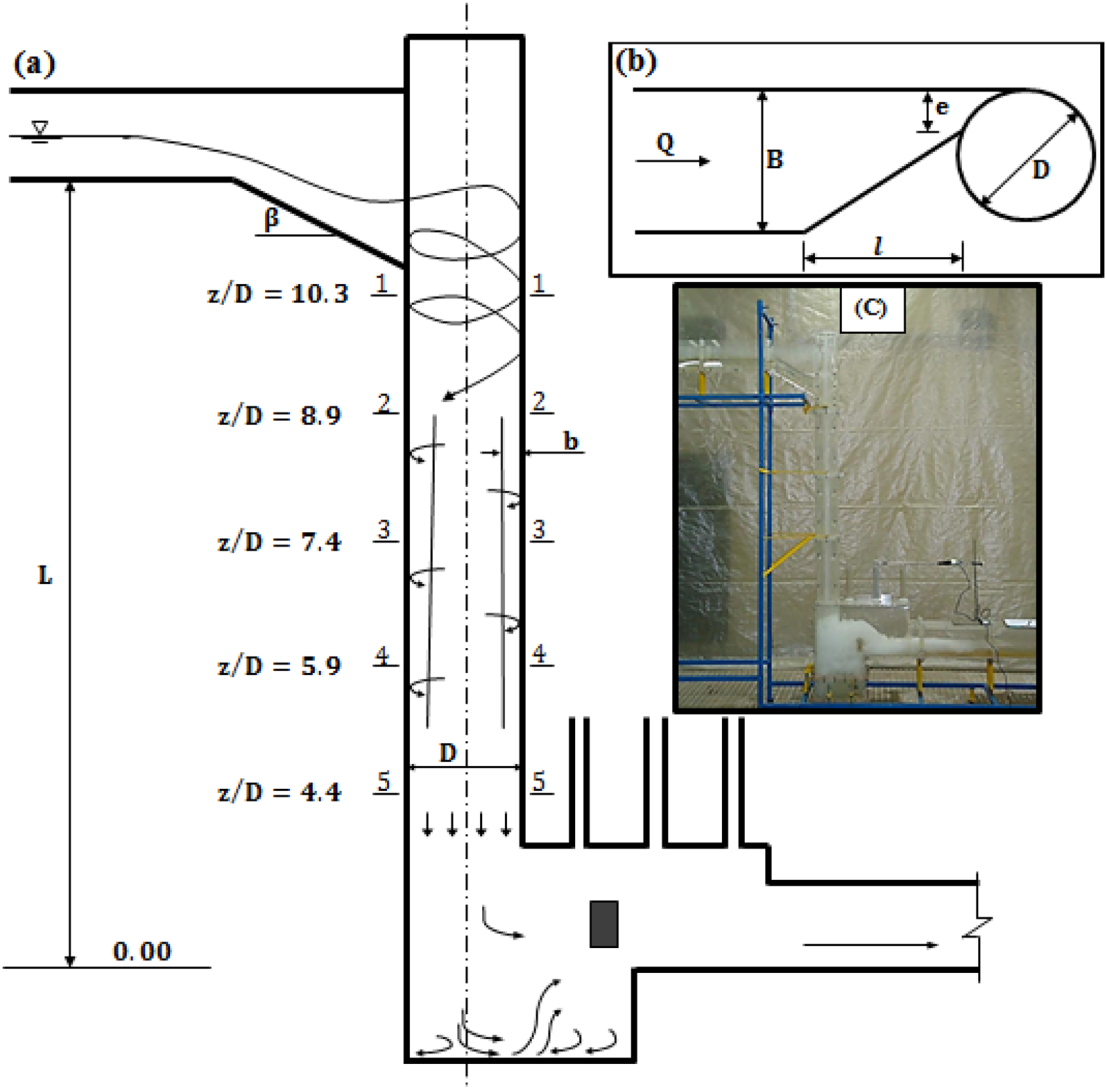
Definition of vortex drop shaft: (**a**) side view, (**b**) tangential inlet plan view, (**c**) photograph of the physical model.

Previous investigations proved that *η*_s_ value in the vertical shaft is computed as^19,25^:

1 $$\eta_{s} = (1 - H_{i + 1} /H_{i} ) \times 100\%$$

where, *H*_*i*_ and *H*_*i*+*1*_ are total energy head at *z*/*D* = 10.3 (section "Introduction") and *z*/*D* = 4.4 (section "Results and discussions") in the vortex drop structure (see Fig. 1), respectively. According to previous experimental investigations^19,24^, the total energy head in each section of the drop shaft is calculated using the following equations:

2 $$H = z + \frac{{V_{z}^{2} }}{2g} + \frac{{V_{t}^{2} }}{2g} + \frac{P\left( r \right)}{{\rho g}}$$

3 $$P\left( r \right) = \mathop \int \limits_{R - b}^{r} \rho \frac{{V_{t}^{2} }}{r}dr = \frac{1}{2}\rho C^{2} \left[ {\frac{1}{{R^{2} \left( {1 - t} \right)^{2} }} - \frac{1}{{r^{2} }}} \right]$$

4 $$C = V_{t} r = \left( {\frac{Q}{e}g} \right)^{\frac{1}{3}} \cos^{\frac{4}{3}} \beta \left( {\frac{D}{2} - \frac{e}{2}} \right)$$

5 $$V_{z} = \frac{Q}{A} = \frac{4Q}{{\pi D^{2} t\left( {2 - t} \right)}}$$

6 $$V_{t} = \frac{4 C}{{\left( {2 - t} \right)D}}$$

in which, *z* = height from zero elevation (origin bed of the outlet channel), *V*_*z*_ = vertical velocity, *V*_*t*_ = tangential velocity, *P*(*r*) = pressure distribution at each section of the drop shaft, *r* = radial coordinate, *R* = radius of the vertical shaft, *D* = diameter of the vertical shaft, *b* = vortex flow thickness, *t* = relative flow thickness (*t* = *b*/*R*), *C* = circulation constant, *Q* = design discharge, *e* = inlet width at the junction of vertical shaft, *g* = gravity of acceleration, and *β* = angle of bottom slope.

The measurement of velocity inside the shaft is performed by replacing the relative thickness of vortex flow (*t*) in Eqs. (5) and (6), vertical and tangential components of velocity are calculated. Therefore, at each cross-section of the vertical shaft, the average values of the relative thickness of the vortex flow are replaced in Eqs. (5) and (6) the instrument shown in Fig. 4. According to Zhao et al.’s^17^ research, a combination of pressure head (referred to the wall pressure where *r* = *R* = *D*/2) and tangential velocity head obtained:

7 $$\frac{{V_{t}^{2} }}{2g} + \frac{P\left( r \right)}{{\rho g}} = \frac{{2C^{2} }}{{gD^{2} \left( {1 - t} \right)^{2} }}$$

In this study, the right-hand of recent equation was used instead of pressure head plus velocity head.

## Dimensional analysis

Over the past decade, results of experimental investigations have discovered that independent variables affecting *η*_s_ values in the vertical shaft are introduced as^19,20,25–27^:

8 $$\eta_{s} = \varphi (Q,B,l,e,\beta ,L,D,f,g,\rho ,\mu ,\sigma )$$

where $\varphi$ = functional symbol, $\rho$ = fluid density, $\mu$ = dynamic viscosity, $\sigma$ = surface tension. According to Fig. 1, other variables are *B* = access channel width, *l* = tangential input length, *L* = drop total height and *f* = friction coefficient. It should be noted that flow depth (*h*) has been measured by piezometers installed bottom of inlet channel and additionally flow average velocity (*V*) at the beginning of tangential inlet is computed by known variables of *Q* and *B*. By considering *Q*, *D*, and $\rho$ as repeating parameters and Eq. (9) is rearranged by using Buckingham theorem:

9 $$\eta_{s} = \varphi \left( {\beta ,f,\frac{Q}{\nu h},\frac{{V^{2} h\rho }}{\sigma },\frac{V}{{\sqrt {gh} }},\frac{B}{D},\frac{l}{D},\frac{e}{D},\frac{L}{D}} \right)$$

where, $R_{r} = Q/h$, $F_{r} = V/\sqrt {gh} ,$ and $W = V^{2} h\rho /\sigma$ are the radial Reynolds number, Froude number, and Weber number of approach flow respectively. In the present study, the radial Reynolds number varies from 213,000 to 360,000 and additionally the Weber number is between 861 and 4066. According to Mulligan et al.’s^28^ investigation, the effects of surface tension and viscosity for vortex flow were ignored due to the fact that *W* and *R*_r_ were greater than 120 and 1000. Therefore, Eq. (10) is re-written as follows:

10 $$\eta_{s} = \varphi \left( {\beta ,f,F_{r} ,\frac{B}{D},\frac{l}{D},\frac{e}{D},\frac{L}{D}} \right)$$

Through this study, *β*, *f*, *B*/*D*, *L*/*D*, and *e*/*D* have constant values which are 29.7°, 0.02, 1.125, 2.18, and 0.25, respectively. Thus, Eq. (10) can be re-writhen as,

11 $$\eta_{s} = \varphi \left( {F_{r} ,\frac{L}{D}} \right)$$

## Physical model layout

**Figure 2:**
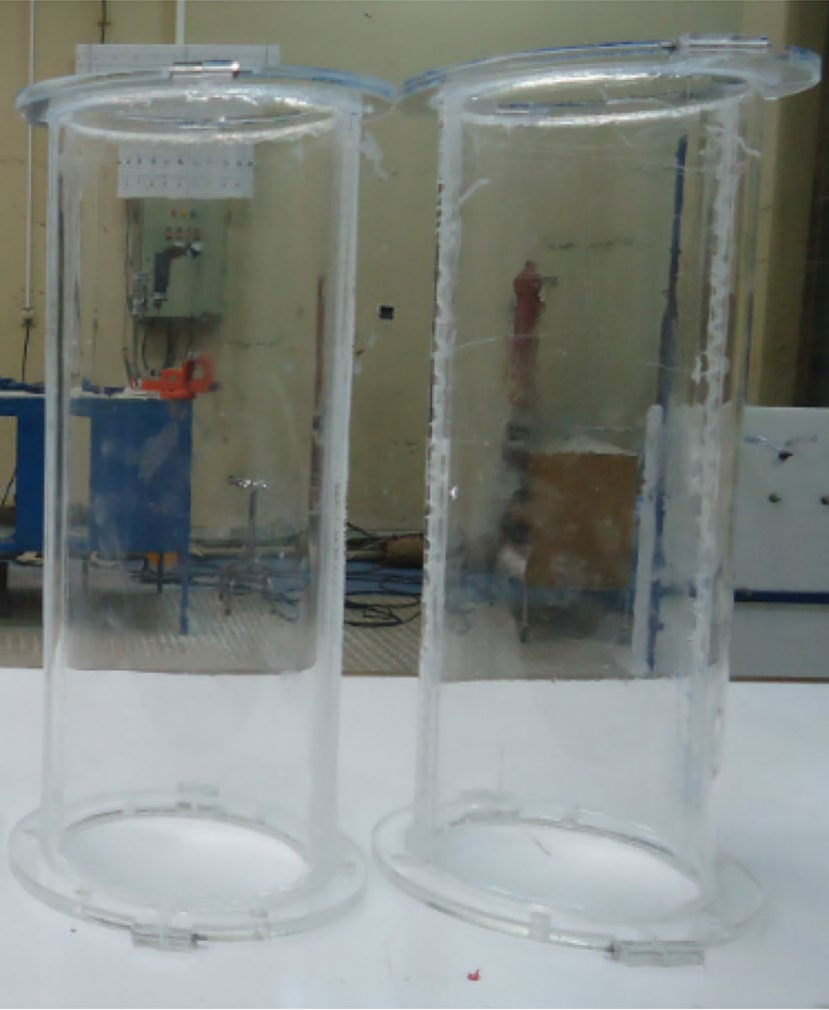
Prefabricated pieces of glass to change shaft height.

**Figure 3:**
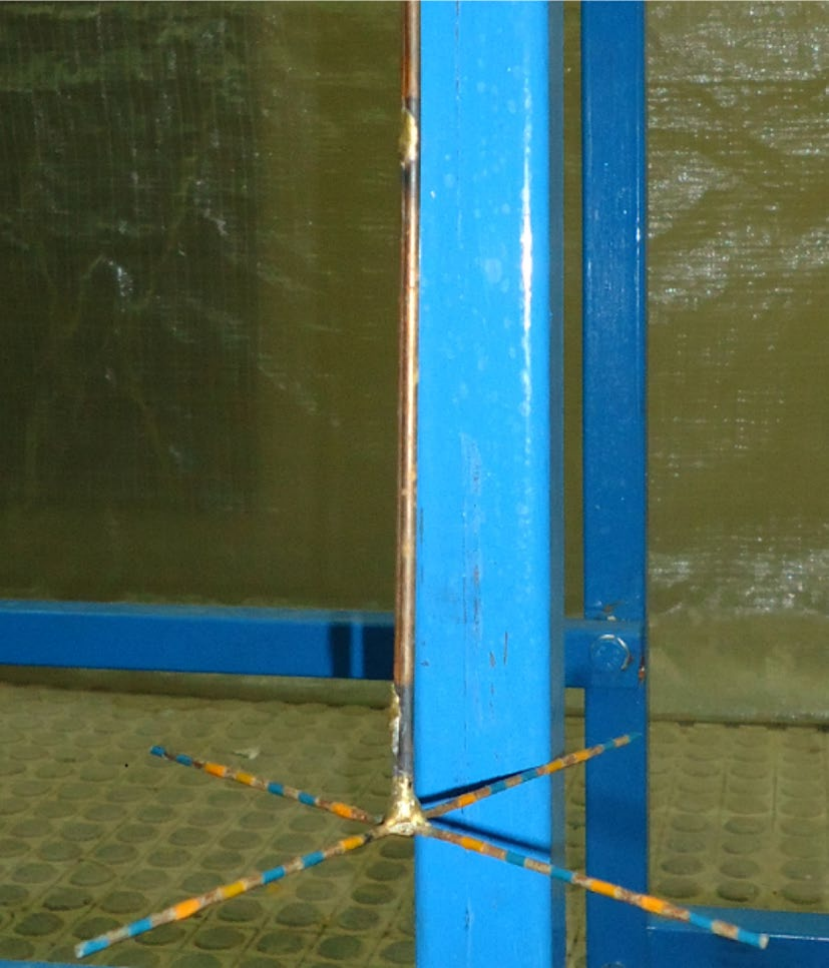
A device manufactured to measure the thickness of the vortex flow.

Physical model of Tehran municipal sewage network vortex structure with a scale of 1:10 is made of transparent Plexiglas. Figure 1c shows a schematic representation of the experimental setup. The physical model consists of rectangular approach channel, tangential inlet, vertical shaft, dissipation chamber, and rectangular outlet tunnel. The stability of vortex flow, proper air circulation, and prevention of flow fluctuations are directly dependent on determining the appropriate diameter for the drop shaft. Therefore, Jain^8^ proposed the relation *D* = *k* × [*Q*_d_^2^/*g*]^0.2^ to determine the diameter of the drop shaft. In recent relation, *k* is the safety factor and $Q_{d}$ is the maximum design discharge. The range *k* = 1–1.25 leads to the economical design of the vortex structure^8,10^. For the present research model with a maximum design discharge equal to 19.4 l/s, the value of *D* = 0.16 m resulted in *k* = 1.22. This research utilizes *L*/*D* ratios of 10, 13, and 16, and additionally two prefabricated pieces with *L*/*D* = 3, as shown in Fig. 2. Moreover, in order to adjust the flow discharge and consequently the Froude number of approach flow (*Fr*) at four levels (1.77, 2.01, 2.18, and 2.32), an electromagnetic flowmeter (MFC 300, Iran Farasanj Abzar, Tehran, Iran) was used with an accuracy of 1%. In this research, air core formed at vertical shaft inlet is evaluated by a self-designed designed four-leg ruler, as depicted in Fig. 3. Using rods with a diameter of 2 mm and creating 5 mm intervals with different colors on each of them, the maximum error in the measurement of the dimeter value of the air core is limited to ± 1 mm.

## Full factorial method

Design of experiments (DoE) is generally used to investigate the effect of the main factors and their interactions on the phenomenon studied^21,23,29^. Regardless of any of the factors in the design of the tests, it is impossible to firmly mention how effective these dimensionless parameters are. According to the literature, the variations of *Fr* and *L*/*D* factors on the loss of flow energy in the vertical shaft have been investigated^14,15,18,19^. This research aimed to study the effects of the main factors on the response by using a full factorial test design. These two factors are among the factors that have been evaluated in the most recent investigations related to vortex structure alone. On the other hand, since the sealing of the vortex structure is the most challenging problem in changing the geometric conditions of a laboratory model, investigation of several geometric factors at the same time will be much tougher. Moreover, with the addition of one or two other factorial tests (taking into account at least three levels for these factors), the number of tests increased from 48 experiments to 144 and 432, respectively. There is no denying the fact that an increase in the number of tests efficiently affects the costs of conducting research.

In this study, a full factorial design has been used to investigate the effect of *Fr* and *L*/*D* factors on *η*_s_ values. Considering 4 and 3 levels for *Fr* and *L*/*D*, a full factorial design includes 12 (4 × 3) possible combinations of these factors. By applying 4 repetitions for each possible combination, 48 (12 × 4) experiments are performed and additionally, Table 1 gives details of the design of the experiments.

**Table 1:** Full factorial design with *η*_s_ (%) values related to each test.

*L/D*	*Fr*			
	1.77	2.01	2.18	2.32
10	33.89	28.21	21.38	11.06
	34.04	28.73	21.51	10.80
	33.79	28.60	21.46	11.06
	34.04	28.60	21.14	12.01
13	50.34	44.73	37.45	22.84
	50.37	44.74	37.24	23.45
	50.48	45.16	37.48	22.98
	50.51	45.23	37.47	23.30
16	62.10	57.84	51.02	36.99
	62.28	58.30	51.69	37.53
	62.29	58.20	51.78	37.60
	62.24	58.17	51.79	37.81

## Results and discussions

### Parametric study

Figure 3 provides readers with information about the meaningful variation of flow energy dissipation efficiency in the vertical shaft (*η*_s_) versus Froude number (*Fr*) for various levels of *L*/*D*. At a quick glance, it is crystal clear that changes of *η*_s_ against *Fr* values at all values of *L*/*D* have gone through downward trends. For all values of *Fr*, *η*_s_ increased with an increase in values of *L*/*D*. For instance, for *Fr* = 1.77, *η*_s_ values augmented from approximately 32% in *L*/*D* = 10 to roughly 62% in *L*/*D* = 16. Additionally, when it comes to a certain value of *L*/*D*, *η*_s_ values plummeted with an increase in *Fr* number. As an example, Fig. 3 demonstrated that, for *L*/*D* = 13,* η*_s_ values declined from about 51% in *Fr* = 1.77 to roughly 25% in *Fr* = 2.32. This investigation follows the 2^nd^ order polynomial regression for the description of variations of* η*_s_ versus *L*/*D* at various levels of *Fr* numbers. As an example, for *L*/*D* = 10, the results of statistical analysis indicated that 2nd order expression provided the most accurate prediction of *η*_s_ values (coefficient of determination [R^2^] = 0.9975) in Supplementary Material (see Fig. S1) when compared with other types of regression equations: linear (R^2^ = 0.932), power (R^2^ = 0.8122), logarithmic (R^2^ = 0.9112), and exponential (R^2^ = 0.8407). All the performances of regression equations for various levels of *L*/*D* were presented in Table 2.

**Table 2:** Results of various curve fitting for understanding variations *η*_s_ of against *Fr.*

*L*/*D*	Type of regression	*η*_s_	*R*^2^
10	Polynomial	−65.14*Fr*^2^ + 225.66Fr–161.5	0.9975
	Exponential	1087.5e^−1.885*Fr*^	0.8407
	Linear	−40.042*Fr* + 106.66	0.932
	Logarithmic	−80.23Ln(*Fr*) + 81.737	0.9112
	Power	331.02*Fr*^−3.755^	0.8122
13	Polynomial	−98.856*Fr*^2^ + 356.12Fr–270.44	0.9924
	Exponential	565.86e−^1.312*Fr*^	0.8183
	Linear	−47.115*Fr* + 136.51	0.8885
	Logarithmic	−94.12Ln(*Fr*) + 106.99	0.8634
	Power	246*Fr*−^2.61^	0.7887
16	Polynomial	−103.66*Fr*^2^ + 380.55*Fr*–286.85	0.9885
	Exponential	300e−^0.852*Fr*^	0.8059
	Linear	−42.28*Fr* + 139.88	0.8524
	Logarithmic	−84.28Ln(*Fr*) + 113.24	0.8246
	Power	174.83*Fr*−^1.694^	0.7755

### Flow pattern in the vertical shaft

**Figure 4:**
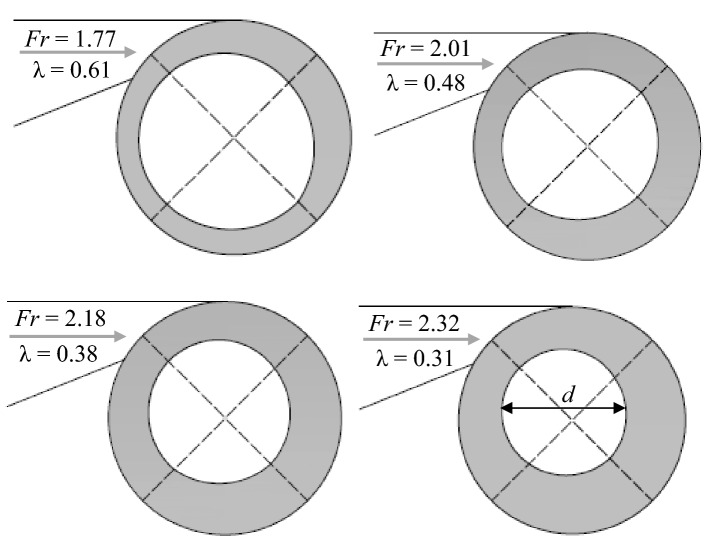
Measurement of air core at the beginning of the vertical shaft.

**Figure 5:**
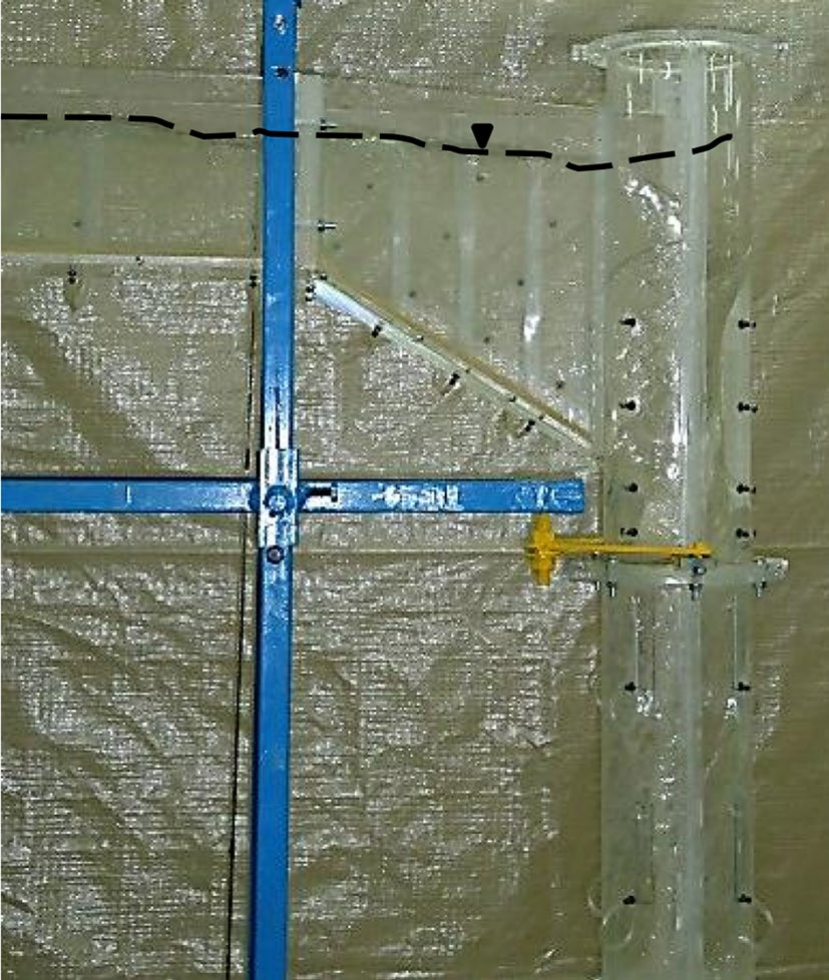
Observed disturbance in the formation of vortex flow for *Q* > 33 l/s.

**Figure 6:**
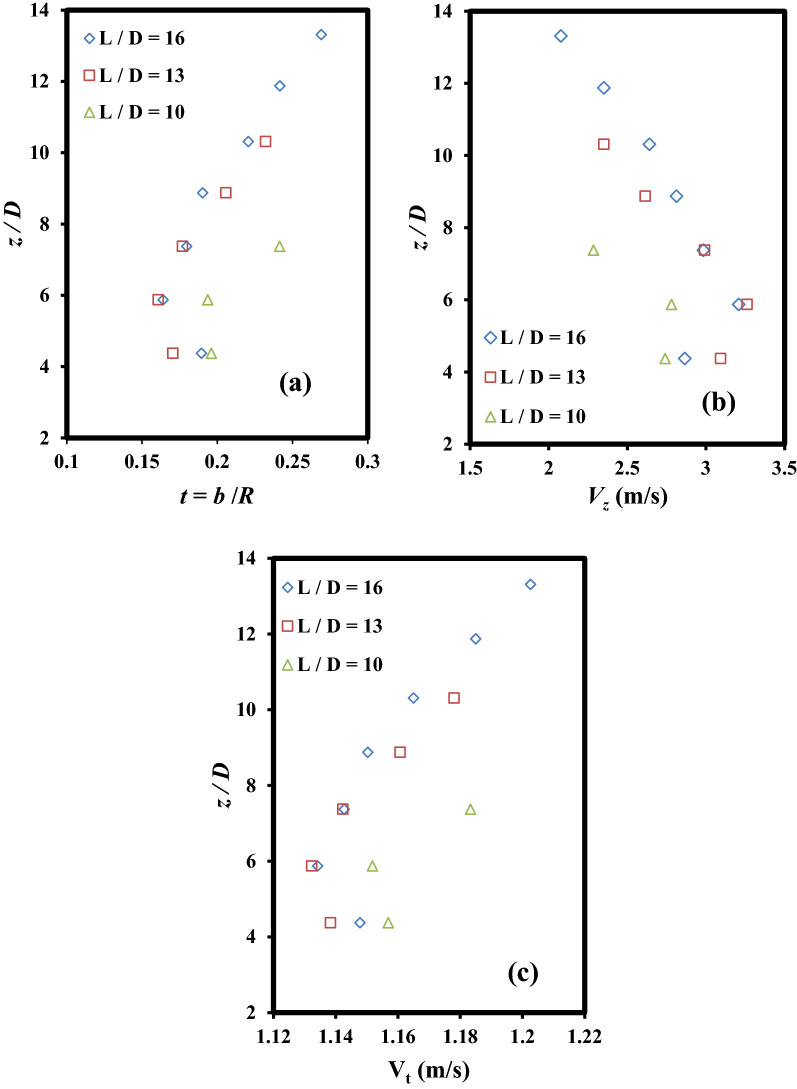
Changes in vortex flow components through the vertical shaft for various values of *L*/*D*: (**a**) relative thickness of vortex flow, (**b**) vertical flow velocity, and (**c**) tangential flow velocity.

The proper formation of the vortex flow along the vertical shaft is influenced by the air core formed at its beginning. According to Yu and Lee^27^ study, the air core area ratio (λ) is approximated by *λ* = *d*^2^/*D*^2^ in which *d* is equal to air core diameter at vertical shaft inlet. As seen in Fig. 4, the vortex flow in the vertical shaft will remain stable if λ ratio is greater than 0.25. The air core measured at the beginning of the vertical shaft for different values of the *Fr* number is shown in Fig. 4. It can be observed that the air core area ratio decreases with an increase in the *Fr* number values (or flow discharge). For *Q* = 1.7*Q*_d_ = 33 l/s, the disturbance of air circulation causes the flow-free surface to become horizontal at the structure inlet and the vortex flow disappears (Fig. 5). Figure 6a–c shows the changes in relative flow thickness (*t*), vertical velocity (*V*_*z*_), and tangential velocity (*V*_*t*_) along the vertical shaft for design discharge and different *L*/*D* levels, respectively. Figure 6a illustrates that, for different levels of *L*/*D* ratio, the mean values of relative flow thickness at higher levels (*z*/*D* > 5.9) decrease rapidly and a slight increase is observed at lower levels. For example, in *L*/*D* = 13, the mean relative thickness values decrease from 0.24 in *z*/*D* = 10.3 to 0.16 in *z*/*D* = 5.9 while for *z*/*D* = 4.4, the mean relative thickness value by slightly increased to 0.17. Figure 6b illustrated that values of vertical velocity had upward trends in the *z*/*D* > 5.9, whereas opposite trends were seen in the lower elevation (*z*/*D* = 4.4–5.9). For instance, for *L*/*D* = 16, values of vertical velocity increase from 2.08 m/s in *z*/*D* = 13.3 to 3.21 m/s in *z*/*D* = 5.9 and then decreased to 2.86 m/s in *z*/*D* = 4.4. Reducing the values of the vertical velocity at the lower levels of the vertical shaft causes a weaker hit of the drop flow inside the dissipation chamber. This weak hit causes the flow of residual energy in the dissipation chamber to be well wasted. Figure 6c demonstrates that, for different levels of *L*/*D* factor, the tangential velocity at higher level (*z*/*D* > 5.9) had downward trends in the *z*/*D* > 5.9 and whereas at lower level, a slight increase is met. For example, in *L*/*D* = 10, the tangential velocity values decrease from 1.18 m/s in *z*/*D* = 7.4 to 1.15 m/s in *z*/*D* = 5.9 and then increases to roughly 1.16 m/s in *z*/*D* = 4.4. Since the tangential velocity for different values of the *L*/*D* factor varies between 1.13 m/s and 1.20 m/s, so from the proximity of the obtained values, the stability vortex flow pattern in the vertical shaft can be concluded. According to the flow pattern presented in Fig. 6a–c, the level of *z*/*D* = 5.9 is the cross section of the vortex flow trend change in the vertical shaft. Table 3 presents the values of flow energy dissipation efficiency in different parts of the vertical shaft (distances among sections 1–2, 2–3, 3–4, and 4–5 in Fig. 1). As can be seen, for *Fr* ≥ 2.01, the energy loss efficiency between sections "Flow energy dissipation efficiency in the vertical shaft" & "Physical model layout" is minimal and for *Fr* = 1.77 the minimum energy loss efficiency occurs between sections "Physical model layout" and "Full factorial method". On the other hand, for all values of *Fr* number (*Fr* = 1.77–2.32), the highest flow energy dissipation efficiency occurs at the lower parts of the vertical shaft (interval between sections "Full factorial method" & "Results and discussions"). According to Table 3, the maximum and minimum efficiencies of flow energy dissipation between different levels of the vertical shaft are 26.43% (*Fr* = 1.77) and 1.88% (*Fr* = 2.32), respectively.

**Table 3:** Efficiency of energy dissipation between both cross sections from vertical shaft.

Fr	${\eta }_{s}^{1-2}\left(\mathrm{\%}\right)$	${\eta }_{s}^{2-3}\left(\mathrm{\%}\right)$	${\eta }_{s}^{3-4}\left(\mathrm{\%}\right)$	${\eta }_{s}^{4-5}(\mathrm{\%})$
1.77	12.21	13.75	11.00	26.43
2.01	10.78	10.16	12.45	21.56
2.18	8.45	7.03	9.62	18.63
2.32	4.99	1.88	5.62	12.64

### Proposed mathematical model of *η*_s_

Analysis of variance is one of the statistical methods used in analyzing laboratory data and expressing the relationship between them. Using this method, the mathematical model governing the results can be extracted by generating data regression and examining the errors. Also, the interaction of laboratory factors in this method will be measured ^29^. According to the variations in the response values in Fig. S1, the initial mathematical model [Eq. (12)] for the flow energy dissipation efficiency in the vertical shaft (*η*_s_) was selected as a second-order polynomial:

12 $$\eta_{s} = c_{0} + c_{1} \left( {F_{r} } \right) + c_{2} \left( {L/D} \right) + c_{3} \left( {F_{r} } \right)\left( {L/D} \right) + c_{4} \left( {F_{r} } \right)^{2} + c_{5} \left( {L/D} \right)^{2}$$

where c_0_ to c_5_ note coefficients are obtained using the least-squares method. The quadratic model adequacy has been evaluated by *R*^2^ and the other statistical parameters were acquired from the analysis of variance (ANOVA). The statistical significance of the model and its terms were obtained *p*-value at 5% level of significance. Values of regression coefficients (*c*_0_ to *c*_5_) and results of the ANOVA for the initial model [Eq. (13)] are shown in Table 4. Accordingly, ANOVA proves that, for the *η*_s_ parameter, the linear and squared effects of factors are highly significant (*p*-value < 0.05). The large *p-*values for the interaction effect of factors (*p*-value > 0.05) presented in Table 4 show that the effects of *Fr* and *L*/*D* factors are just factors affecting energy loss in the vertical shaft. Additionally, since only the terms with *p*-value < 0.05 remain in the model, the interaction effect of *Fr* and *L*/*D* factors is removed from the initial model and then *c*_3_ = 0 is considered. After removing the interaction effect of *Fr* and *L*/*D* factors from the initial model, the final reduced quadratic model was obtained by re-analyzing the ANOVA as follows:

13 $$\eta_{s} = - 318.2574 + 320.7605\left( {F_{r} } \right) + 7.4360\left( {L/D} \right) - 89.2151\left( {F_{r} } \right)^{2} - 0.1028\left( {L/D} \right)^{2}$$

**Table 4:** ANOVA of full quadratic model [Eq. (12)].

Source	Coefficient estimate (${c}_{i}$)	Sum of squares	Degree of freedom	Mean square	F-value	*p*-value	Significant
Model	–	10,730.91	5	2146.18	1418.16	< 0.0001	*
Intercept	−328.3180	–	1	–	–	–	
${X}_{1} : Fr$	325.6207	3912.81	1	3912.81	2585.51	< 0.0001	*
${X}_{2} : L/D$	8.2099	6465.25	1	6465.25	4272.11	< 0.0001	*
${X}_{1}{X}_{2}$	−0.3739	1.69	1	1.69	1.12	0.2963	
${{X}_{1}}^{2}$	−89.2151	426.65	1	426.65	281.92	< 0.0001	*
${{X}_{2}}^{2}$	−0.1028	9.13	1	9.13	6.03	0.0183	*
Residual	–	63.56	42	1.51	–	–	

The results of the analysis of variance of the reduced quadratic model with some statistical indicators are presented in Table 5. The following results can be obtained from the ANOVA analysis:

i. The final model with *p*-values < 0.0001 indicates that the model is statistically significant with a confidence level of 99.99%.
ii. According to Table 5, R^2^ = 0.994 indicates that 99.4% of the response changes can be described.
iii. In the reduced models, the $R_{Adj}^{2}$ value is always equal to or less than *R*^2^. In this way, it can be said that the reduced model has a reasonable number of mathematical terms^30^. From Table 5, the quality of the reduced model is shown in a reasonable number of mathematical terms with $R_{Adj}^{2} = 0.993$ and R^2^ = 0.994.
iv. The statistical index of $R_{Pred}^{2}$ shows the model's ability to predict a set of new data. According to Table 5, the high values of this index ($R_{Pred}^{2} = 0.992$) show the ability to final model [Eq. (13)] in this regard.
v. The coefficient of variance (COV) is the ratio of standard deviation to the mean-value of the measured response (as a percentage). The COV is the reproducibility factor of a mathematical model. On the other hand, since models with COV < 10% are models that can be reproduced, the final model with COV = 3.21% also benefits from this ability.
vi. The adequate precision (AP) represents the ratio of the difference in the predicted response value of the model with the average value of predictive error. AP ≥ 4 shows the appropriate performance of the mathematical model in predicting response values. From Table 5, $AP = 132.936$ indicates the capability of the final model [Eq. (13)].

**Table 5:** ANOVA of Eq. (13) with its statistical indicators.

Source	Coefficient estimate (${c}_{i}$)	Sum of squares	Degree of freedom	Mean square	F-value	*p*-value	Significant
Model	–	10,729.22	4	2682.31	1767.54	< 0.0001	*
Intercept	−318.2574	–	1	–	–	–	
${X}_{1} : Fr$	320.7605	3912.81	1	3912.81	2578.40	< 0.0001	*
${X}_{2} : L/D$	7.4360	6535.67	1	6535.67	4306.78	< 0.0001	*
${{X}_{1}}^{2}$	−89.2151	426.65	1	426.65	281.15	< 0.0001	*
${{X}_{2}}^{2}$	−0.1028	9.13	1	9.13	6.01	0.0183	*
Residual	–	65.25	43	1.52	–	–	
Model statistical indicators							
${R}^{2}$		0.994		COV (%)		3.21	
${R}_{Adj}^{2}$		0.993		AP		132.936	
${R}_{Pred}^{2}$		0.992					

**Figure 7:**
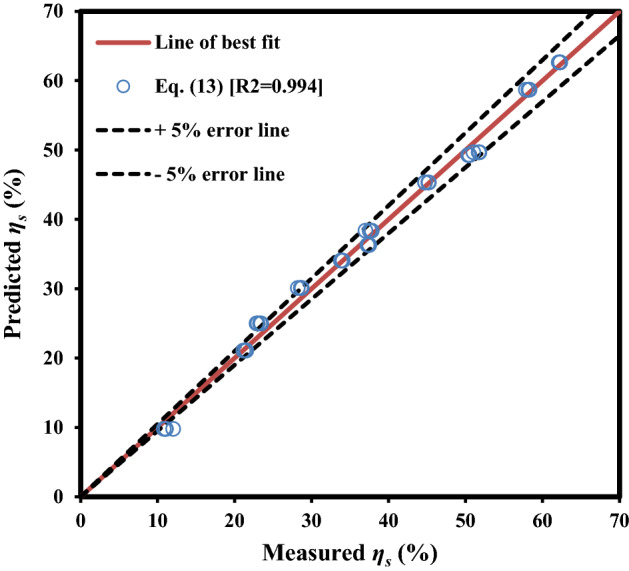
The measured *η*_s_ versus predicted ones by Eq. (13).

In addition to the mentioned items, the graphical performance of the final model [Eq. (13)] is shown in Fig. 7 by providing a scatter plot of the measured* η*_s_ data versus the predicted data ones. This plot indicates an adequate agreement between observed *η*_s_ values and estimated ones by Eq. (13).

### Effect of main factors on ***η***_s_

According to *p*-value statistical index presented in Table 5, the effects of *Fr* and *L/D* on* η*_s_ variable are shown in Supplementary Material (see Fig. S2). From Fig. S2a, variations of *η*_s_ versus Froude numbers *Fr* had a quadratic downward trend for *L*/*D* = 13. Fig. S2a illustrated that the effect of the second-order term of the Froude numbers *Fr* on the* η*_s_ variable is considerable. This conclusion can be obtained from ANOVA (Table 5). The *p*-values of *Fr*^2^ term is less than 0.0001, showing that the *Fr*^2^ term is significant in the final model. Although according to Table 5, the effect of first and second-order terms of *L*/*D* factor on response values is statistically significant. Fig. S2b shows that changes in response values are more influenced by the first-order term of *L*/*D* factor. In addition to the mentioned item, Fig. S2b shows that the value of* η*_s_ increases with an increase in *L*/*D* value.

### Qualitative performance of the mathematical model

**Figure 8:**
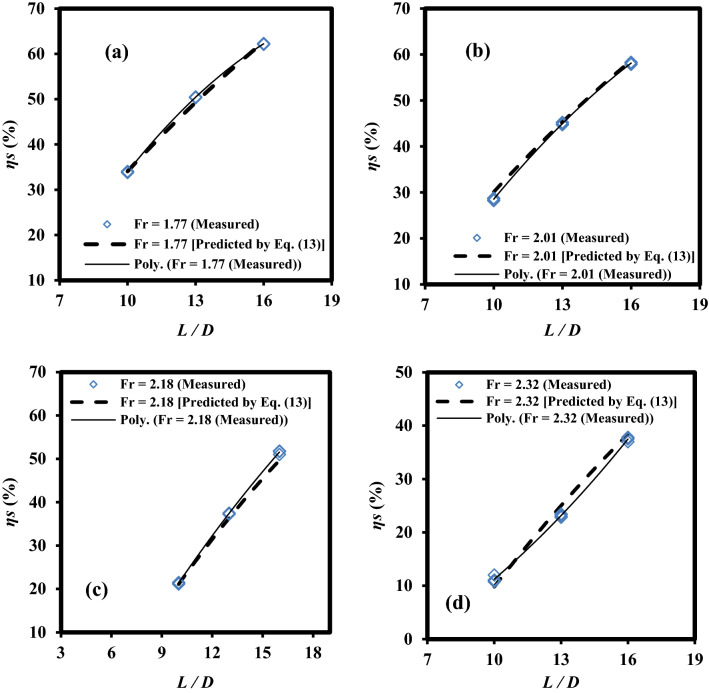
Variations of *η*_*s*_ values versus *L*/*D* factor for various values of *Fr* factor: (a) *Fr* = 1.77, (b) *Fr* = 2.01, (c) *Fr* = 2.18, and (d) *Fr* = 2.32.

In this section, the qualitative performance of the final model [Eq. (13)] in predicting *η*_s_ values for different levels of *Fr* and *L*/*D* factors is investigated. Figure 8a–d illustrates the trend of variations in the measured and predicted values of *η*_s_ versus the *L*/*D* factor for *Fr* = 1.77, 2.01, 2.18, and 2.32. More specifically, Fig. 8 illustrated that variations of *η*_s_ against *L*/*D* at disparate levels of *Fr* number had an upward trend. For instance, at the level of *Fr* = 1.77, Fig. 8a demonstrated that *η*_s_ values were gently on the rise, increasing from roughly 33% in *L*/*D* = 10 to approximately 62% in *L*/*D* = 16. At the same level of *L*/*D* values, the efficiency of energy dissipation in the shaft decreased with an increase in Fr values. As a noticeable example, for *L*/*D* = 13, *η*_s_ values surged from 50% in *Fr* = 1.77 to roughly 45% in *Fr* = 2.01 as indicated in Fig. 8a, b, respectively. Then, *η*_s_ plummeted to roughly 38% and 20% in *Fr* = 2.18 and 2.32, as seen in Fig. 8c, d, respectively. In this study, the second-order polynomial was fitted to the experimental data in Fig. 8.

**Figure 9:**
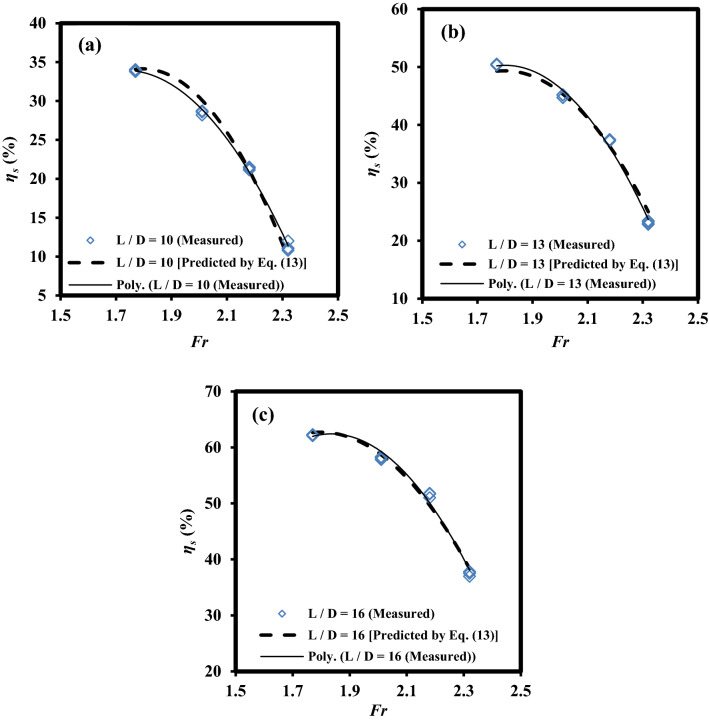
Variations of *η*_*s*_ values versus *Fr* factor for various values of *L*/*D* factor: (**a**) *L*/*D* = 10, (**b**) *L*/*D* = 13, and (**c**) *L*/*D* = 16.

Figure 9 indicated the robustness of Eq. (13) in order to describe variations of* η*_s_ values versus *Fr* number for all levels of *L*/*D*. As seen in Fig. 9, changes in *η*_s_ values against *Fr* values follow a downward trend. For instance, the performance of Eq. (13) indicated that, for *L*/*D* = 16, the predicted* η*_s_ values declined from roughly 62% in *Fr* = 1.77 to about 38% in *Fr* = 2.32. Moreover, at the same level of *Fr* number,* η*_s_ values rose with an increase in *L*/*D*. As an example, for *Fr* = 2.32,* η*_s_ values increase from roughly 12% in *L*/*D* = 10 to approximately 38% in *L*/*D* = 16.

According to Figs. 8 and 9, the maximum absolute difference of *η*_s_ values (maximum difference between measured and predicted values) with a negligible amount of 2.21% shows that Eq. (13) has an acceptable performance in predicting *η*_s_ values.

### Comparison of the present results with literature

In this section, the results of experimental research have been compared with relevant literature. The present study employed a broad range of flow discharge, Froude number, and *L*/*D* ratio, compared to the previous investigations. As seen in Table 6, many studies (e.g., Vischer and Hager^14^, Jain and Kennedy^15^) conducted experiments with the unchanged values of *L*/*D* ratios and consequently, any mathematical models were not inevitably extracted owing to the limit range of effective parameters. This research provided an empirical equation including *L*/*D* and *Fr*. Additionally, *L*/*D* ratio used by Zhao et al.’s^19^ research was in the range of the present investigation *L*/*D* (10–16). By the way, the energy efficiency obtained from the results of Zhao et al.’s^19^ experiments was 34% which was in the range of the present study (10.80–62.29%). As a major merit compared with literature, the present results can be generalized for further ranges of effective factors (i.e., *Fr* and *L/D*) which are not observed in this study. In this way, existing interaction between effective factors and efficiency of energy loss can be obtained without the performance of the experiments when experts applied effective factors whose limits are within the range of the present tests parameters.

**Table 6:** Comparison of the present results with relevant literature.

Authors	*Q* (m^3^/s)	*L*/*D*	*η*_S_ (%)
Vischer and Hager^14^	140	50	85
Jain and Kennedy^15^	90	100	90
Jeanpierre and Lachal^18^	88	9	62
Zhao et al.^19^	0.051	14	34*
This study	0.0097–0.0271	10–16	10.80–62.29

*For half of the length of a drop shaft with *L* = 14*D.*

### Consent to Publish

All the authors give the Publisher the permission of the authors to publish the research work.

## Conclusion

This study investigated the experimental model of urban wastewater vortex structure. Flow energy loss in the vertical shaft has been studied to provide a mathematical model in order to investigate various effects of contributing parameters (*Fr* and *L*⁄*D*) on the flow behavior in the vertical shaft of the vortex structure. In this way, the main findings of this research can be summarized as follows:

- For *Q* ≥ 1.7 *Q*_*d*_, the air core formed at the beginning of the vertical shaft was destroyed and while the air circulation is disturbed, the free surface of the flow in the inlet channel is almost horizontal and the formation of vortex flow is disturbed.
- The relative thickness values of the vortex flow along the vertical shaft for all *L*/*D* factor levels start with a relatively significant decreasing trend and reach almost the constant value at low elevations.
- Reducing the vertical velocity values for all levels of *L*/*D* factor at levels which was close to the dissipation chamber causes the drop flow to enter this section with less impact and improves the energy dissipation efficiency in this section.
- Very close values of tangential velocities at different levels of the vertical shaft for all *L*/*D* factor levels indicated that the vortex flow was kept stable along the vertical shaft.
- Analysis of variance showed that the interaction of *Fr* and *L*/*D* factors on the values of the flow energy dissipation efficiency (*η*_s_) was not statistically significant. Therefore, it can be said that the trend in variations of the values of the *η*_s_ versus one factor (or *Fr* and *L*/*D*) at different levels of another factor remained unchanged.
- The results showed that the effects of the first and second-order terms of *L*/*D* ratio on response values were significant. It was also shown that the effect of first-order term on changes in response values was more important and the trend of changes did not have a certain trend.
- Response values variations versus the *Fr* factor were chiefly affected by the second-order term of this factor and the trend of changes in response values followed a quadratic manner.
- The flow energy dissipation efficiency in the vertical shaft (*η*_s_) plummeted when *Fr* had an increasing trend. In contrast, *η*_s_ and *L*/*D* ratio had inverse dependence.
- The flow energy dissipation efficiency in the vertical shaft (*η*_s_) was obtained between 10.80 and 62.29%.
- A quadratic polynomial equation has been proposed in order to understand the physical meaning of the *η*_*s*_ values against *Fr* and *L*/*D* along with *R*^*2*^ = 0.994. The proposed equation was reliable for *Fr* = 1.77–2.32 and *L*/*D* = 10–16.
- The level of *z*/*D* = 5.9 was introduced as a cross-section in which trend of variations in vortex flow properties (i.e., tangential velocity, vertical velocity, and relative flow thickness) in the vertical shaft was observed.

## Supplementary Information

Supplementary Information.

## Data Availability

The data are not publicly available due to restrictions such their containing information that could compromise the privacy of research participants. Contact the corresponding author to request data.
