# Supplementary material for: Experimental evaluation of the energy dissipation efficiency of the vortex flow section of drop shafts
Source: Sci Rep. 2023 Jan 30;13:1679. doi: 10.1038/s41598-023-28762-2 (PMC9886980; doi:10.1038/s41598-023-28762-2)
Supplement: Supplementary file 1 — Supplementary Information. [file 41598_2023_28762_MOESM1_ESM.docx]

In this Supplemental Materials, there are two figures as follows:

Fig.S1. Variations of *η*_s_ against *Fr* values for various values of *L*/*D*

**(b) Actual Factors**

***Fr* = 2.18**

Fig.S2. Main factors effects on *η*_s_: (a) influence of *Fr* on *η*_s_ and (b) influence of *L*/*D* on *η*_s_.
